# Irritable Bowel Syndrome: Yoga as Remedial Therapy

**DOI:** 10.1155/2015/398156

**Published:** 2015-05-06

**Authors:** Vijaya Kavuri, Nagarathna Raghuram, Ariel Malamud, Senthamil R. Selvan

**Affiliations:** ^1^Vivekananda Yoga Research Foundation, Norwalk, CA 90650, USA; ^2^Swami Vivekananda Yoga University (SVYASA), Bangalore, Karnataka 560019, India; ^3^White Memorial Medical Center, Los Angeles, CA 90033, USA

## Abstract

Irritable bowel syndrome (IBS) is a group of symptoms manifesting as a functional gastrointestinal (GI) disorder in which patients experience abdominal pain, discomfort, and bloating that is often relieved with defecation. IBS is often associated with a host of secondary comorbidities such as anxiety, depression, headaches, and fatigue. In this review, we examined the basic principles of *Pancha Kosha* (five sheaths of human existence) concept from an Indian scripture *Taittiriya Upanishad* and the pathophysiology of a disease from the Yoga approach, *Yoga Vasistha's Adhi* (originated from mind) and *Vyadhi* (ailment/disease) concept. An analogy between the age old, the most profound concept of *Adhi-Vyadhi*, and modern scientific stress-induced dysregulation of brain-gut axis, as it relates to IBS that could pave way for impacting IBS, is emphasized. Based on these perspectives, a plausible Yoga module as a remedial therapy is provided to better manage the primary and secondary symptoms of IBS.

## 1. Introduction

Irritable bowel syndrome (IBS) is defined as “abdominal pain or discomfort that occurs in association with altered bowel habits over a period of at least three months, in the absence of any detectable organic cause” [1, 2]. It can be classified as either diarrhea-predominant (IBS-D), constipation-predominant (IBS-C), or IBS with alternating stool pattern (IBS-M (mixed)) [3]. IBS is the most prevalent functional gastrointestinal (GI) disorder in the world [4]. The prevalence varies according to country and age, with worldwide prevalence rates ranging from 9 to 23% and in USA from 10 to 15% [5]. IBS is the most common disorder diagnosed by gastroenterologists and accounts for up to 12% of total visits to primary care providers [4]. It can affect people of all ages but is more likely to occur between 20 and 45 years of age. More women suffer from this disorder with an incidence ratio of 2 : 1 between females and males [6]. It is suspected that the changing hormones in the female menstrual cycle may be a reason for this disparity [7]. IBS has a significant impact on health care use and costs, which is heightened due to the imprecise nature of its diagnosis and treatment. The aggregate cost, direct and indirect, of treating IBS in the United States has been estimated at $21 billion or more annually, excluding prescription and over the counter drug costs [8, 9]. In this review, we have made an attempt to compare the viewpoint of ancient Indian scriptural understanding of IBS with that of mind-gut model of conventional medical science that offers a strong basis to combine Yoga as a vital therapeutic modality in the holistic approach to the management of IBS.

**Figure 1:**
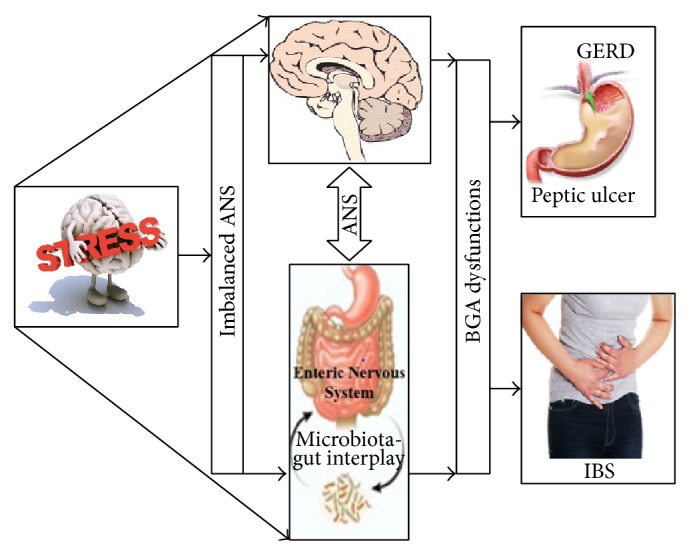
Role of stress in the exacerbation/development of stress. Stress-induced pathophysiological consequences of the disruption of brain-gut axis. Exposure to stress affects autonomic nervous system, causing an imbalance that results in the disturbance of brain-gut axis (BGA). This leads to the development of different diseases of the gastrointestinal tract, including gastroesophageal reflux disease (GERD), peptic ulcer disease, and irritable bowel syndrome (IBS) (source of various images: stress, http://www.1stclassmed.com/; brain, http://lifehacker.com/; stomach/intestines, http://www.rechildrens.org/blog/gut-brain-axis/; GERD, http://www.highlandersurgicalassociates.com/; holding the stomach, http://www.paleoibs.com/).

*Comorbidities of IBS*. IBS is called a functional disorder because no structural, biochemical, or infectious etiology has been found and is a disorder of motor and sensory functions of the GI tract [10]. Whitehead et al. observed a strong association of psychiatric disorders in 94% of IBS patients pointing to the role of psychological factors in the etiology of IBS [11]. Headache, fibromyalgia, and depression were commonly found in a study of about 100,000 individuals with IBS [12]. A systematic review found that IBS occurs in 51% of chronic fatigue syndrome patients and 49% of fibromyalgia patients [12]. Evidence supports an important role of stress in IBS patients, particularly in altering brain-gut interactions, resulting in development or exacerbation of IBS [13]. Symptoms of IBS seem to worsen during periods of stressful events [14], as illustrated in Figure 1. Diagnosis of IBS also includes identifying key stressors/triggers such as emotional, physical, or sexual abuse and psychological distress including anxiety and depression [2]. Thus, it appears that IBS is a manifestation of “derailing of the brain-gut axis” [15].

*Conventional Management of IBS and Its Limitations*. Conventional management of IBS primarily involves dietary modifications, medications, and psychotherapy. Medications may consist of anticholinergics, stool softeners, and laxatives such as dicyclomine for abdominal cramps, lubiprostone for IBS-C, and loperamide for IBS-D [16]. Although the conventional modalities of treatments produce favorable outcomes, there have been reported side effects. Loperamide has been shown to be effective in slowing down the movement of the gut, decreasing the number of bowel movements, and makes the stool less watery, but side effects of nausea, cramping, and constipation have been reported [17]. Antispasmodics like dicyclomine improve symptoms of IBS and reduce pain, but side effects may include drowsiness, dry mouth, blurred vision, or inability to urinate [1]. Fiber is often recommended as a dietary change to reduce global IBS symptoms, but the clinical data to date are less clear. Notably, soluble fiber can lower GI symptoms in IBS-C but was not able to reduce pain perception in IBS patients [18, 19]. As the conventional medical treatments are not always effective in producing a satisfactory clinical response outcome, more than 50% of people who suffer from IBS turn to alternative medicine [20]. Since IBS often results from a combination of physical and stress-related factors, a treatment approach addressing both body and mind would be most appropriate. In this context, IBS sufferers seek alternative remedial approach such as probiotics, herbal remedies, acupuncture, and Yoga [20]. Even though psychotherapy like hypnosis and cognitive behavioral therapy (CBT) have been shown to be effective in improving quality of life and reduction in pain, IBS patients have to continue therapy sessions at frequent intervals to maintain the benefits [19]. This is considered to pose financial burden and time constraint for the patients.

## 2. Ancient Indian Approach of Managing IBS

According to Ayurveda, an ancient Indian medical science developed around 3,500 BC, a disorder called “*Grahani*” can be correlated to IBS.* Grahani* can be a syndrome with alteration of stool either in solid or in liquid form. The main causative factor ascribed for this disorder is the malfunctioning of* Agni* (digestive fire). According to Caraka,* Agni* is the most important factor and it regulates all metabolic processes and maintains the overall health and zeal. If* Agni* is impaired, body functions are impaired [21].* Agni* is deranged by excessive fasting, eating during indigestion, overeating, irregular eating, and intake of unhealthy and contaminated foods [22].* Agni* is also deranged by emaciation, faulty adaptation to place, time, and season, and suppression of natural urges. Stress levels (*mano-udvega*) influence one's eating habits and lifestyle. Treatment in Ayurveda mainly focuses on increasing* Agni* that promotes the healthy functioning of the gut, with various herbal preparations like* chitrakadi vati* (*Plumbago zeylanica*),* bilwa adi vati* (*Aegle Marmelos*), and Basti, a colon cleansing enema, with medicated oil or ghee (clarified butter) and herbal decoction. Meditation,* pranayama*, and* Yogasanas* are beneficial for the mind in reducing the stress. Yoga is a prescribed remedy to treat* Grahani* as a part of Ayurveda treatment [23]. According to the scriptures of Ayurveda and Yoga, the health at the physical level can only be achieved by balanced functioning of all aspects of an individual (*samatolanam*/homeostasis) and, hence, it is important to treat the person as a whole (“mind, body, and breath”).

## 3. Yoga

Yoga, a traditional “mind-body-breath” discipline, was derived from India about 3,500 BC. The word Yoga, in Sanskrit, is “*yuj*” meaning to unite the mind, body, and spirit [24]. Yoga encompasses mental and physical discipline to help in personal transformation that leads to perfect health as envisioned by WHO. Yoga practitioners are totally immersed in the practices with an awareness and command over every movement of the body and synchronizing it with the breath. This gives them a feeling of self-control and calmness leading to relaxation and health. The popularity of Yoga is evident with a prevalence of 8.7% Yoga practitioners in 2012 within the USA that is equivalent to 20 million adults, and women practitioners outweigh males by a ratio of almost 4 : 1 [25]. There is a vast amount of literature available, listing the benefits of Yoga, among which, a few studies are alluded to, including IBS and Yoga, in this review.

### 3.1. Yoga and Physical Well-Being

Flexibility is an important component of health-related physical fitness and well-being [26]. Adequate range of motion, lower back, and shoulder flexibility are extremely important for day to day performance. Several studies have shown that Yoga improves the flexibility of the body both internal and external. In 1986, Ray et al. [27], have shown that six-month practice of Yogic asanas increased trunk, hip, neck, and shoulder flexibility in middle-aged men whereas physical exercises had no such effect. Two recent studies of chronic lower back pain patients also concluded that Yoga increased spinal flexibility and quality of life better than physical exercise [28, 29]. Yoga's emphasis on developing body awareness and physical discipline supports the adoption of healthy dietary and exercise habits and thus potentially could influence the management of IBS symptoms. Several research studies have emphasized that Yoga makes one feel “more connected” to their bodies (internal and external organs) and promotes a positive body experience and a sense of well-being [30, 31]. Ducrotté [32] suggested that Yoga postures targeting the lower abdomen would help in relieving the symptoms of IBS by enhancing bioenergy circulation in and around the intestines.

### 3.2. Yogic Breathing and Autonomic Balance

Yoga practices offer the possibility of reducing inappropriate activation of the autonomic nervous system (ANS). Clinical trials on IBS patients have shown abnormalities in autonomic function and psychological profiles. Some studies have shown that there is an increased sympathetic activity in IBS patients. Using spectral analysis of heart-rate variability in 54 subjects (18 IBS patients and 36 healthy controls), it was reported that IBS patients had significantly increased sympathetic activity compared to healthy controls, whereas there was no difference in parasympathetic activity between these two groups [33]. In a study conducted by Waring et al. [34] on 69 female subjects (39 with IBS and 30 healthy controls), sympathetic excess in IBS patients was observed during stimulation (handgrip exercise and orthostatic test) when compared to the healthy controls. In contrast, Punyabati et al. [35] reported increased parasympathetic reactivity and elevated levels of anxiety in 65 IBS patients.

The relaxing and calming effects of* Savasana* (total relaxation) and* pranayama* (breath control) have been widely studied and reported. The effects of these practices provide a short-term “time out” from stress and also by creating positive physiological changes in the whole body through modulating the nervous system [36].* Sudarshan Kriya* is a simple rhythmic breathing technique with specific natural rhythms of the breath, harmonizing the body, mind, and emotions. It is shown to alleviate symptoms of anxiety, depression, and stress-related ailments [37]. Slow and deep breathing techniques could be used to minimize physiologic responses to stress by increasing the parasympathetic response [38]. Breathing through right nostril was shown to increase oxygen consumption, an indicator of increased metabolism and the sympathetic activity, whereas left-nostril breathing led to parasympathetic shift [39]. A two-month study of 21 male adult IBS-D patients (Yoga group = 9; conventional group = 12), with Yoga intervention of a few postures and “voluntary” regulated right-nostril breathing, to be practiced at home, showed that both groups had positive changes over time in general autonomic functions, bowel symptoms, state anxiety scores, and gastric motility (electrogastrography (EGG)) amplitude. The Yoga group showed significant improvements in autonomic symptom score, bowel symptom score, state anxiety, and physical flexibility whereas the control group had significant improvements in resting EGG amplitude [40]. Though beneficial effects of Yoga were reported in this study, the sample size lacked female patients in whom IBS is more prevalent. As the evidence is clear that IBS patients could have sympathetic/parasympathetic dominance, balancing the autonomic nervous system should be a focus, rather than emphasizing on right nostril breathing which could increase the sympathetic activity. This emphasizes an urgent need of a systematic clinical trial, testing the most practical Yoga module, for the better management of IBS.

### 3.3. Yoga and Psychological Well-Being

A large number of studies confirm that Yoga enhances general psychological well-being. A moving meditation called “Cyclic Meditation” based on stimulation and relaxation [41] showed reduced oxygen consumption compared to resting in supine position and reduced sympathetic activity and increased parasympathetic dominance [42]. A subsequent study showed reduced stress and increased quality of sleep after 23 minutes of Cyclic Meditation [43]. Rosary (Ave Maria) and certain Yoga mantras when recited at specific frequencies (six times a minute) improved physiological and psychological well-being and also an increase in the synchronicity of cardiovascular rhythms were observed in 23 healthy volunteers [44]. Yoga is also shown to reduce anxiety and symptoms of depression and enhance quality of life. Two studies have reiterated that three and four months of Yoga practice had significantly improved the subjective well-being and quality of life [45, 46].

Yoga intervention for IBS adolescent patients that consisted of four weeks of daily home practices showed lower levels of functional disability, less use of emotion-focused avoidance, and lower anxiety than the control group. A 10-minute video was provided to the children to practice at home for four weeks. A 65% practice was reported. Most of the children also reported that when in pain, they did not practice and, other times, could not find time to practice [47]. Even though the study reported improvement, a close supervision of Yoga sessions was deficient. A recent study on 51 adolescent and young adult IBS patients using Iyengar Yoga for 6 weeks, 90 minute class twice a week, reported positive changes in IBS symptoms, sleep, fatigue, and psychological distress when compared to control group with usual care [48]. Iyengar Yoga is characterized by great attention to detail and precise focus on body alignment, with the use of “props” such as cushions, benches, blocks, straps, and sand bags which the IBS patients might not appreciate. Timing (how long to hold a position), technique (precision of the body alignment), and sequence of the asanas are very important for this style of Yoga, which could be a challenge for these patients.

### 3.4. Yoga and Quality of Life

In an eight-week intervention of mindfulness meditation study, meditators were shown to have higher activation in left-sided frontal lobe that is associated with positive feelings such as joy, happiness, compassion, and lower levels of anxiety, when compared with the control group of nonmeditators [49]. A recent study investigated the effects of Brain Wave Vibration (BWV), a meditation involving rhythmic movements of the head, neck, and body practiced with related Yoga style exercises for eight weeks. Thirty-one healthy adults were assessed in total for both groups. BWV group showed better mood, sleep, mindfulness, health, and well-being compared to controls that participated in Yoga style exercise without the movements [50].

An earlier study [51] assessed the role of stress in 50 medical students, in which the Yoga group practiced one hour Yoga, three times a week, for a month and the control group did reading/writing for the same duration. Yoga group showed improvements in various parameters such as sense of well-being, feeling of relaxation, improved concentration, self-confidence, improved efficiency, better interpersonal relationship, increased attentiveness, lowered irritability levels, and optimistic outlook in life. Similar findings were reported in the Yoga practitioners by later studies. In a survey, 61 Yoga practitioners had a positive outlook on life, and happiness within, when compared to 135 who were non-yoga-practitioners [52]. Rocha and colleagues [53] concluded that regular Yoga practice (6 months) can improve aspects of cognition and quality of life for healthy individuals. Slow and deep breathing for six weeks improved cognition and general well-being and increased parasympathetic activity as compared to controls [54]. In yet another survey, 1,045 Yoga practitioners reported to have improved energy, happiness, social relations, and sleep [55].

Taken together, Yoga practices enhance physical flexibility, energy levels, and quality of life with improved psychological disorders. It also increases self-awareness, and happiness within, and also makes one feel relaxed and rejuvenated. Therefore, Yoga practices are considered much more useful in IBS patients at various levels to better manage their symptoms.

## 4. *Pancha Kosha*-Balanced Approach of Yoga Therapy (PK-BAYT)

Yoga is being explored for potential health benefits in the recent past, with the advent of increased awareness for prevention of diseases. Most of the scientific evidences available to date used parts of Yoga, that is, postures, or breathing exercises or meditations to show the benefits of Yoga. In fact, Patanjali's Ashtanga Yoga encompasses a variety of mind-body-breath practices:* Yama* (rules for virtuous living in the external world),* Niyama* (the moral injunctions for healthy living for oneself),* Äsanas* or postures,* Prānāyāma* or breath control, and four stages of meditation (*Pratyahara*,* Dhärana*,* Dhyänä*, and* Samädhi*) that enables mastery over the mind. Thus, Yoga has the potential to bring about physical, mental, emotional, and spiritual well-being in the practitioners. The concepts of* Pancha Kosha* and* Adhi/Vyadhi* are very helpful in explaining the modern day psychosomatic illnesses or disorders and in creating appropriate therapeutic Yoga modules. We have explored the ancient Indian literature in understanding the Yoga concept of illness and pathophysiology of IBS from the texts (*Adhi/Vyadhi* from Yoga* Vasistha*,* Pancha Kosha*, the five layered human existence, from* Taittiriya Upanishad*) in providing a comprehensive Yoga module as a remedial therapy for IBS.

**Figure 2:**
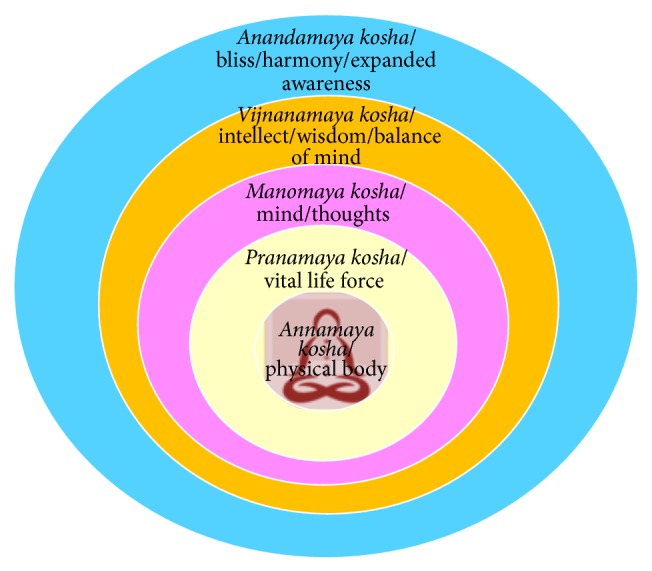
Schematic representation of* Pancha Kosha*, five layers of human existence.

*Taittiriya Upanishad* [56] described* Pancha Kosha* concept of five intertwined layers of human existence (Figure 2). The first four layers are interacting and interdependent layers on the background of the fifth layer of bliss in which there are perfect balance, harmony, and health. Accordingly, the goal of human life is to transcend to the 5th layer of bliss, beyond all disturbing mental processes, by performing each and every duty/action in blissful awareness without unwanted reactions to chronically demanding situations of life. This is the key to achieving total peace that leads to perfect health.

### 4.1. *Annamaya Kosha* (Physical Body)

*Annamaya kosha* is the physical frame of the body and is the grossest of all five layers, representing the anatomy that is a conglomeration of subtle particles (such as electrons) that go on to form highly organized systems. This is nurtured by the nutrients, the food we eat. A healthy body is the key to maintain homeostasis of different systems within.

### 4.2. *Pranamaya Kosha* (Vital Life-Force)

The* pranamaya kosha* ensures the harmonious functioning of these organs by the physiological processes.* Prana* (vital life-force) is the basic life energy inside and outside the body. A uniform flow of this life-force to each and every cell of the physical body (*annamaya kosha*) keeps it healthy. If there is a disturbance in the flow of* Prana* to any organ, it can lead to dysfunction of that organ at the physical body level.

### 4.3. *Manomaya Kosha* (Mind)

*Manomaya kosha* is the mental and emotional library of the human system. According to Bhagavadgita [57] (ch II, verses 60–62), the psychological stresses (emotions) begin as uncontrolled rewinding surge of thoughts in this layer. Meditation is the tool to manage the stresses from mind level.

### 4.4. *Vijnanamaya Kosha* (Intellect)

*Vijnanamaya kosha* is the discriminating faculty (inner mind, conscience) which guides the* manomaya kosha* constantly to get mastery over the basic instincts perceived by the sense organs. In a recent review article, Deshmukh explains that, in* Dhyänä*-Yoga (Sanskrit word for meditation), there is a natural sense of well-being with self-understanding, spontaneous joy, serenity, freedom, and “self-fulfillment” [58]. The secret for happiness, according to scriptures, is conquering the mind through knowledge. Once the mind is calm, it operates with logic and is able to deal with situations in a balanced way that leads to healthy functioning of the physical body.

### 4.5. *Anandamaya Kosha* (Bliss)

*Anandamaya kosha* is the most subtle layer in the array of the five layers of human existence. This layer is not bound by time or space and is devoid of emotions, a state of total silence, complete harmony, and perfect health [59]. Happiness is within us, a state of inner silence.* Taittiriya Upanishad* describes the process in which a student realizes that all layers of our existence emerge from* anandamaya kosha* [60]. It leads to the insight that happiness is within us and “each one of us” in our causal state is “*Ananda*” (bliss) embodied. Practicing Yoga with complete awareness and involvement of mind-body-breath is the technique to achieve a healthy body and a blissful state. This underscores that only with the awareness and involvement of “mind-body-breath” one could experience the therapeutic benefits of Yoga.

## 5. Yogic Perspective of Pathophysiology of Illness

“*Yoga Vasistha*” [61] provides Yoga perspective of the pathophysiology of diseases. According to this text (ch II, verses 709–723), psychosomatic diseases originate from the mind, percolate to subtle energy called the vital life-force, and settle in the physical body, inflicting damage to the weakest organ affecting the physiology and functioning of those organs. In the case of IBS, the target organ is the gastrointestinal tract.

There are two kinds of diseases:* Adhija* (originated by mind) and* Anadhija* (non-stress-related)* vyadhis*.* Adhis* are twofold:* Samanya* (ordinary) and* Sara* (essential).* Samanya*/ordinary diseases could be termed as life style noncommunicable diseases since these are produced during the interactions (mental conflicts) with the world.* Sara adhija vyadhi* is the essential disease of being caught in the birth-rebirth cycle that can be understood in modern terms as congenital diseases. The* sara adhija vyadhi* does not cease until knowledge of the self (*atma jnana*) is attained [62].* Anadhi vyadhis* are non-stress-related diseases like those due to injury, infection, and toxins and could be treated with any available modern and Ayurvedic medicines.

**Figure 3:**
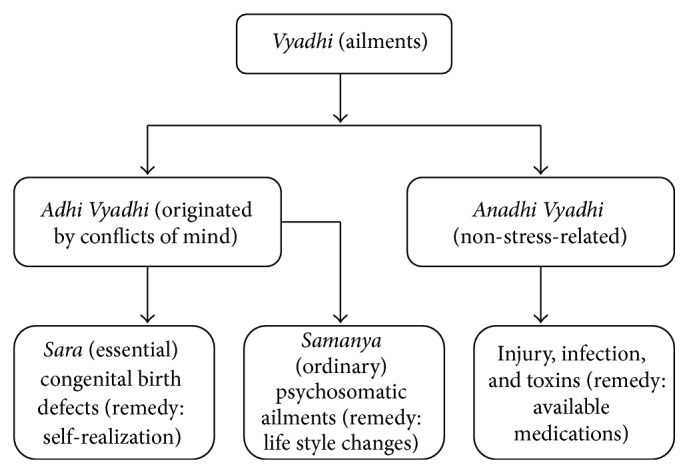
Yogic classification of diseases.

In the layer of bliss, a human being is the healthiest, with harmony and balance of all sense organs. At the* vijnanamaya kosha*, there are disturbances in the mind but are channeled in the right direction [59]. The imbalances start at the* manomaya kosha*, mind layer. Likes and dislikes, happiness and sorrow, and love and hatred are some of the dualities that start governing the human actions, often directed by emotions and not the intellect. When these imbalances intensify, they result in mental conflicts called “*Adhis*” [61]. Yoga looks at these mental conflicts as uncontrolled speed of thoughts in the mind. The consequence is wrong life style; four components of the lifestyle, diet, lack of exercise, dire habits (smoking, alcohol, and uncontrolled desires), and emotional stress, are all traceable to mind. With the repetition of mental conflicts comes a habituated response of anxiety, depression, or anger ultimately affecting the functioning of various systems.  Figure 3 depicts schematically yogic understanding of ailments.

Yoga can bring about changes in the* Samanya* type of* Adhis* corresponding to the modern day mind-body diseases. When the mind is agitated constantly, the vital life-force (*varistha prana*) in the body is affected by imbalances in the breath and causes dysfunction in the five channels of life-force. The five channels of prana are: (1)* Prana*, controls the functioning of the heart and lungs and all the activities in the chest region like breathing, swallowing, and circulation of blood; (2)* Apana*, controls the function of the excretory and reproductive organs and hence is responsible for all downward activities like urination, defecation, and menstruation; (3)* Samana*, activates and controls proper digestion and is responsible for balancing* Prana* and* Apana*; (4)* Udana*, is responsible for all upward activities such as belching and vomiting; and (5)* Vyana*, is responsible for all activities on the periphery like nerve impulses and cellular activity in all cells and gives extra boost to other four channels when required [63, 64].

**Figure 4:**
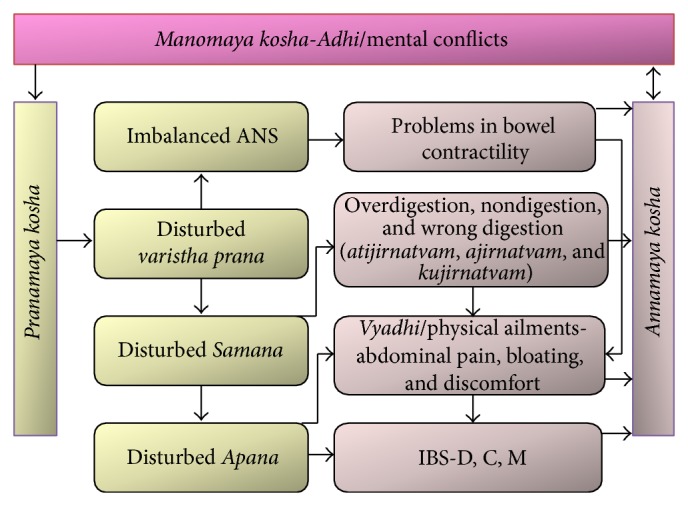
Schematic representation of* Adhi* becoming* Vyadhi* in the context of IBS. Conflicts in the mind disturb the vital life-force (*varistha prana*) that affects* Samana* (responsible for digestion) and disturbed autonomic nervous system. When* Samana* is disturbed, there is an imbalance in* Apana* in case of IBS patients. Disturbed* varistha prana* causes imbalance in the ANS and bowel contractility issues. All these disturbances are thought to be the onset of IBS (diarrhea, constipation, or both mixed).

Due to the imbalance in the vital life-force (*varistha prana*), caused by the mental conflicts, the autonomic nervous system is disturbed and it might result in heightened bowel contractility. Of the five life-forces, the most important for the healthy functioning of the body is “*Samana*,” a life-force that is responsible for digestion and balancing two other major life-forces, “*Apana*” and “*Prana*.” When “*Samana*” is disturbed, the food ingested cannot be digested properly. This leads to overdigestion (*atijirnatvam*), nondigestion (*ajirnatvam*), or wrong digestion (*kujirnatvam*) and thus improperly digested food when settles in the body leads to diseases [65]. If “*Samana*” becomes dominant, it leads to gastroesophageal problems, including epigastric pain, hyperacidity and gastric reflux.* Ajirnatvam* and heightened sympathetic activity (disturbed ANS) results in IBS-C. Disturbed “*Samana*” causes an imbalance in “*Apana*,” and excess* Apana* along with parasympathetic dominance manifests as IBS-D (*atijirnatvam*) or IBS-M (*kujirnatvam*) (Figure 4; [66]). In other words, heightened parasympathetic activity results in IBS-D and heightened sympathetic activity results in IBS-C. Increased peristaltic waves lead to diarrhea and excess saccular contractions due to sympathetic activity leads to constipation [67, 68]. Remarkably, this ancient concept of* Adhi/Vyadhi* appears very similar to the modern scientific pathway of stress-induced brain-gut axis dysfunction as outlined in Figure 1.

## 6. Yoga as Remedial Therapy for Management of IBS

Long standing persisting anxiety, anger, and depression (stresses) that lead to mind body illnesses are the habituated pattern of responses characterized by this rewinding violent loop of thoughts. Hence the remedy is to slow down (*prashamana*) these looping thoughts that would have gathered enough power to disturb the grosser layers of* pranamaya* and* annamaya koshas* [62]. According to Patanjali, mastery over the modifications of the mind is Yoga [69].* Pancha Kosha*-Balanced Approach of Yoga Therapy (*PK*-BAYT) utilizes mind-body-breath healing techniques to balance and harmonize the disturbances of psychosomatic ailments at all the five levels of our existence. Physical exercise is a key component to maintain health at* annamaya kosha*. Physical movements with breathing are emphasized at this* kosha*, breathing practices to expand lung capacity and loosening practices to stretch and relax the muscles and loosen the joints; Yoga postures can provide the strength and endurance to the physical body and provide deep relaxation to the body and mind. Varying postures are used based on the specific ailment.* Kriyas* (cleansing techniques) are also used in enhancing the treatment to the physical layer. At* pranamaya kosha*, various breathing practices are used to expand the lung capacity and to balance the breath in order to impact the functioning of the cells by calming the nervous system. The first step is to master the* Asana* or posture to control the body and sit comfortably for a length of time to practice* pranayama* [70]. This helps to achieve varying breathing exercises and get relief from the dysfunctional parts of our body.

At* manomaya kosha,* various levels of meditation that includes* Pratyahara* (withdrawal of mind from the objects of sense perception),* Dhärana *(focusing on a single thought),* Dhyänä* (effortless flow of a single thought in the mind), and* Samädhi *(merger in the object of meditation) [69] are used to calm down the mind. Notional corrections and self-analysis to enhance better judgment are some techniques that are helpful in balancing the* vijnanamaya kosha*. At* anandamaya kosha*, action-in-relaxation is practiced to experience bliss continually.

Johannesson and colleagues observed that any moderate physical activity ranging from 20 to 60 minutes, three times a week, improved symptom severity of IBS when compared to controls that did not do physical exercise [71]. In this study, duration and type of the activity was not constant for all subjects.

In light of these studies, and with the knowledge of ancient scriptures, various aspects of traditional Yoga (*Patanjali* and* Yoga Vasistha*) are used to create a Remedial Yoga Module for the management of IBS symptoms. A brief summary of Yoga practices that would be most applicable for IBS patients with the goals of balancing each* kosha* is given in Table 1. Yoga sessions were standardized to create consistency among patients and instructors. A distinct and concise Yoga module for IBS is given as follows that is very practical to follow under the supervision of a certified Yoga instructor and adherence to it.

*Summary of Remedial Yoga Module for IBS*

*Names of Yoga Practice*

1. Starting affirmation (A-U-M, sound three times and OM, three times);
2. Breathing practices:
  - hands-stretch breathing,
  - *Vyaghrasana*/tiger pose breathing,
  - *Shashankasana*/moon pose breathing,
  - *Padottanasana*/straight leg raise breathing (supine position, both legs);
3. * Savasana*/instant relaxation;
4. * Sithilikarana Vyayama*/loosening exercises:
  - forward and backward bending,
  - side bending,
  - twisting,
  - *Pavanamuktasana Kriya*/Wind relieving pose with leg rotation;
5. * Savasana*/quick relaxation;
6. * Asanas*/postures (standing, sitting, prone, and supine):
  - *Ardhakati cakrasana*/half-waist wheel pose,
  - *Ardha cakrasana*/half wheel pose,
  - *Padahastasana*/hands to feet pose,
  - *Trikonasana*/triangle pose,
  - *Parivritta trikonasana*/revolved triangle pose,
  - *Vrikshasana*/tree pose,
  - *Vakrasana*/half spinal twist,
  - *Pascimottanasana*/seated forward bend pose,
  - *Bhujangasana*/cobra pose,
  - *Shalabhasana*/locust pose,
  - *Sarvangasana*/shoulder stand,
  - *Viparitakarani* with wall support/legs up the wall,
  - *Matsyasana*/fish pose;
7. * Savasana*/deep relaxation;
8. * Kriya*/cleansing and pranayama/regulated breathing:
  - *Kapalabhati*/forceful exhalation,
  - *Uddiyana Bandha and Agnisara*/abdominal lock and rigorous movement of abdomen,
  - *Vibhagiya Svasana*/sectional breathing,
  - *NadiShuddhi pranayama*/alternate nostril breathing,
  - *Sitali*/cooling* pranayama*,
  - *Sitkari*/cooling* pranayama*,
  - *Bhramari*/M-chanting;

(9) *Dhyana*/meditation:
  - * Nadanusandhana*/Yoga of sound (A-U-M),
  - OM meditation (OM);
(10) Closing affirmation, OM three times.

Given the gradual personalized training pace, people with discomfort also can follow this Yoga module that is designed to cover the whole body with particular focus on the abdominal region. Stretching and twisting of the abdomen with awareness that offers deep local rest to the abdominal viscera are emphasized; this would help in alleviating distension and pain in the abdomen.* Uddiyana Bandha* (abdominal lock) and* Kriyas* [72] like* Basti* (colon cleansing),* Agnisara* (rigorous movement of the abdominal muscles), and* Viparitakarani kriya* (cleansing breath in half shoulder stand) are considered very helpful for IBS patients.* Prānāyāma* relevant to IBS, in harmonizing the autonomic nervous system, such as slow sectional breathing, alternate nostril breathing, and cooling* Prānāyāma* have been included. A-U-M* meditation* and OM* meditation* are included to calm down the mind. The Yoga module that we have come up with is different from others for the reasons that all the patients will practice for an hour, under the supervision of a certified Yoga instructor, it is easy to follow, and it includes targeted practices of the abdomen. The practices designed are easy to perform from easy breathing practices to abdomen-stretching, twisting postures, and resting the body and systems with relaxation to give relief to the symptoms. Since there is an instructor in the class, the patients are actively guided and monitored. This module emphasizes “mind-body-breath” connection more than physical alignment, timing, or sequencing of* asanas* as it would be challenging for beginners of Yoga. Hence, this would be a highly practical module for IBS patients to pursue and adhere to.

## 7. Conclusion

In this review, we attempted to provide a distinct synthesis of the Eastern (India) scriptural concepts of IBS as of mental origin, and thus Yoga approach of managing IBS as implemented in our randomized clinical trial (Clinical Trial Number: ISRCTN42102754; manuscript in preparation) with our design of concise an hour Remedial Yoga Module. The Yoga module comprises the essential components of traditional Yoga, that is, “postures, breathing, and meditation” that can be easily practiced by most patients, with least complications to enhance the therapeutic impact on IBS. Importantly, Yoga modality is envisioned from the cost effectiveness in managing IBS and its related comorbidities like anxiety, depression, and fatigue.

## Figures and Tables

**Table 1:** *Pancha Kosha*-Balanced Approach of Yoga Therapy (*PK*-BAYT).

Layers/koshas	Natural state	Altered state	Yogic remedy
Physical (*annamaya*)	Relaxed	Muscular tensions, abdominal pain, bloating, constipation, and diarrhea	Stimulate the body with various loosening practices, relax with postures, and cleanse the internal organs with cleansing techniques

Life force (*pranamaya*)	Slow rhythmic breath	Haphazard breathing, wrong direction, wrong quantities, and imbalances	Slow down the breath with breath control and balance the flow of vital life force

Mind (*manomaya*)	Calm state	Mental agitations, stress, anxiety, and depression	Calm down the mind with meditation and devotion

Intellectual (*vijnanamaya*)	Wisdom	Wrong perceptions, distorted cognition, and lack of discrimination	Notional corrections and self-inquiry for better judgment and increased self-confidence

Bliss (*anandamaya*)	Harmony	Disharmony and unhappiness	Action in relaxation, to experience the bliss continuously, selflessness, and happiness within
